# Blue-Light Filtering Spectacle Lenses: Optical and Clinical Performances

**DOI:** 10.1371/journal.pone.0169114

**Published:** 2017-01-03

**Authors:** Tsz Wing Leung, Roger Wing-hong Li, Chea-su Kee

**Affiliations:** 1 School of Optometry, The Hong Kong Polytechnic University, Hung Hom, Kowloon, Hong Kong SAR; 2 School of Optometry, University of California, Berkeley, California, United States of America; Universidade do Minho, PORTUGAL

## Abstract

**Purposes:**

To evaluate the optical performance of blue-light filtering spectacle lenses and investigate whether a reduction in blue light transmission affects visual performance and sleep quality.

**Methods:**

*Experiment 1*: The relative changes in phototoxicity, scotopic sensitivity, and melatonin suppression of five blue-light filtering plano spectacle lenses were calculated based on their spectral transmittances measured by a spectrophotometer. *Experiment 2*: A pseudo-randomized controlled study was conducted to evaluate the clinical performance of two blue-light filtering spectacle lenses (BF: blue-filtering anti-reflection coating; BT: brown-tinted) with a regular clear lens (AR) serving as a control. A total of eighty computer users were recruited from two age cohorts (young adults: 18–30 yrs, middle-aged adults: 40–55 yrs). Contrast sensitivity under standard and glare conditions, and colour discrimination were measured using standard clinical tests. After one month of lens wear, subjective ratings of lens performance were collected by questionnaire.

**Results:**

All tested blue-light filtering spectacle lenses theoretically reduced the calculated phototoxicity by 10.6% to 23.6%. Although use of the blue-light filters also decreased scotopic sensitivity by 2.4% to 9.6%, and melatonin suppression by 5.8% to 15.0%, over 70% of the participants could not detect these optical changes. Our clinical tests revealed no significant decrease in contrast sensitivity either with (95% confidence intervals [CI]: AR–BT [–0.05, 0.05]; AR–BF [–0.05, 0.06]; BT–BF [–0.06, 0.06]) or without glare (95% CI: AR–BT [–0.01, 0.03]; AR–BF [–0.01, 0.03]; BT–BF [–0.02, 0.02]) and colour discrimination (95% CI: AR–BT [–9.07, 1.02]; AR–BF [–7.06, 4.46]; BT–BF [–3.12, 8.57]).

**Conclusion:**

Blue-light filtering spectacle lenses can partially filter high-energy short-wavelength light without substantially degrading visual performance and sleep quality. These lenses may serve as a supplementary option for protecting the retina from potential blue-light hazard.

**Trial Registration:**

ClinicalTrials.gov NCT02821403

## Introduction

Blue light is short-wavelength electromagnetic radiation (400–500 nm) in the visible spectrum (400–780 nm) that carries the highest amount of energy per photon. It has drawn increasing attention due to a hypothesis suggesting that blue light has the potential to induce photochemical damages to the retina.[1–5] Data from animal and *in vitro* studies suggest that in an ageing eye, the accumulation of lipofuscin (in particular its major retinoid fluorophore A2E) within the retinal pigment epithelium makes the retina even more vulnerable to high-energy blue radiation, leading to cell apoptosis.[6–9] This process may explain why excessive sunlight exposure associates with age-related macular degeneration (AMD),[10–12] the third leading cause of worldwide blindness,[13] though other hypotheses have been advanced.[14–16]

The sun and artificial light sources, including LED (light-emitting diode) light bulbs and fluorescent light tubes, are the primary sources of blue light, emitting the amount of blue light that approaches the international exposure limit.[17] With the increasing popularity of blue-rich LED-backlight display devices, such as mobile smartphones, ultraportable tablets, and computer screens, our eyes are exposed to more blue light than in the past. There has been a surge of new ophthalmic aids including intraocular lenses[18] and spectacle lenses designed with a rationale to protect the eyes from potential photochemical damages. These lenses claim to use filtering materials or surface coatings to reduce the spectral transmittance of short-wavelength blue light. However, one key challenge when using these products is finding the balance between effectively reducing blue-light hazards without compromising the essential visual functions in daily life, i.e., there is a pressing need to determine the potential benefits of these blue-light filtering ophthalmic devices.[19]

While excessive blue light is theoretically harmful, adequate blue light is necessary for normal visual function. For example, blue light plays an important role in colour discrimination[20] and night vision[21]. The S-cone and rod photoreceptor cells, which are responsible for these visual functions, reach their maximum sensitivity under blue and blue-green environments. While blue-light filtering intraocular lenses have been widely tested in laboratory and clinical studies,[22–31] the optical characteristics and clinical performance of similar products for spectacle lenses remain unclear. In addition, daytime exposure to blue light regulates the human internal circadian (24-hour) biological clock, stimulating the brain to stay awake during the day by inhibiting melatonin secretion.[32] It has been proposed that avoiding blue light during the day could disturb the natural sleep-wake cycle and hence negatively affect sleep quality.[31, 33]

There were two goals in this study: firstly, to measure the spectral transmittance of five commercially available blue-light filtering lenses and calculate the relative changes in phototoxicity, scotopic sensitivity, and melatonin suppression; secondly, to evaluate two of the five blue-light filtering lenses in a pseudo-randomized controlled study involving 80 computer users. The latter study included evaluation of colour vision, contrast sensitivity, night vision, and sleep quality of the participants after wearing blue-light filtering lenses for one month.

## Materials and Methods

### Optical evaluation of blue-light filtering lenses

Five representative blue-light filtering spectacle lenses (refractive index = 1.6, refractive power = 0D) were evaluated (“BlueControl”, Hoya, Japan; “BlueProtect”, Zeiss, German; “Crizal Prevencia”, Essilor, France; “StressFree” and “Noflex”, Swiss Lens, Hong Kong). Their spectral transmittances, *T(λ)*, within the wavelength *(λ)* range of 300 to 780 nm, were measured (step size = 5 nm) using a LAMBDA 650S UV/Vis spectrophotometer (Perkin-Elmer, USA), with the lens geometric center aligned with the measuring axis. The spectral transmittance data was used to calculate the relative changes in phototoxicity (*ΔB*), scotopic sensitivity (*ΔV’*), and melatonin suppression (*ΔC*) using the following equations as published in several ophthalmic optics standards:

$$\Delta B=1-\frac{\int_{300}^{780}T(\lambda )B(\lambda )d\lambda }{\int_{300}^{780}B(\lambda )d\lambda }$$

$$\Delta V\prime =1-\frac{\int_{300}^{780}T(\lambda )V\prime (\lambda )d\lambda }{\int_{300}^{780}V\prime (\lambda )d\lambda }$$

$$\Delta C=1-\frac{\int_{300}^{780}T(\lambda )C(\lambda )d\lambda }{\int_{300}^{780}C(\lambda )d\lambda }$$

where *B(λ)* denotes the blue-light hazard function, *V’(λ)* denotes the scotopic luminous efficiency function, and *C(λ)* denotes the circadian efficiency function. More details of these three functions can be found in the guidelines published by the International Commission on Non-Ionizing Radiation Protection (ICNIRP)[34], the Commission Internationale de I’Eclairage (CIE)[35] and the German Institute for Standardisation (DIN)[36], respectively. Rather than predicting the biological changes, these calculations can serve as a reference for future lens design, because the optical characteristics of these spectacle lenses vary between companies. Also, there is currently no strict guideline for this type of special lens product.

We also measured the front surface reflectance of each blue-light filtering spectacle lens in order to identify whether they reduce blue-light transmittance through their blue-filtering anti-reflection coating (reflect blue light) or yellow/brown-tint filtering materials (absorb blue light).

### Evaluations of blue-light filtering lenses on essential visual functions and sleep quality

**Fig 1:**
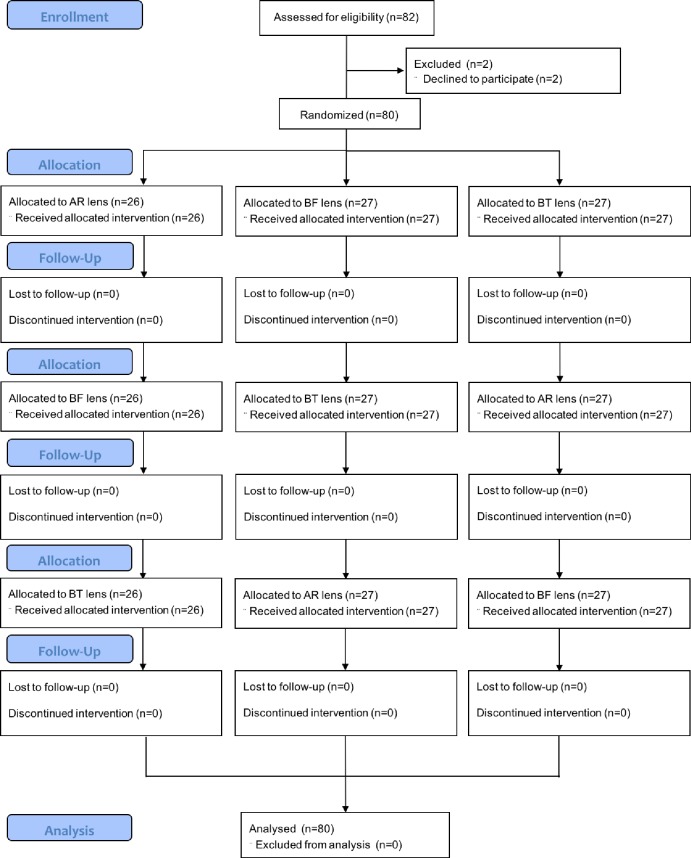
The flowchart for this pseudo-randomized controlled clinical trial.

In the second study, a single-masked pseudo-randomized controlled clinical trial was conducted to evaluate whether blue-light filtering spectacle lenses affected visual performance and sleep quality. A double-masked study as originally planned was not possible because an investigator could differentiate the three lenses due to prior experience in ophthalmic dispensing. Lenses from the Swiss lens company were chosen because they offered blue-light filtering lenses of different optical designs: (1) “Stressfree”: a clear lens with blue-filtering anti-reflection coating on the front surface (BF; blue-light transmittance: 82.2%); and (2) “Noflex”: a brown-tinted lens (BT; blue-light transmittance: 77.5%). A clear lens with conventional anti-reflection coating (AR) from the same company was used as a control (blue-light transmittance: 90.0%). All lenses blocked UVA, UVB, and near-UV (380–400 nm) rays (transmittance <0.2%) and fulfilled the ISO requirements for category 0 ophthalmic lenses, i.e., transmittance of visible light over 80%.[37] Fig 1 shows the flowchart of this clinical trial.

Since visual function begins to deteriorate in middle age,[38–40] this age-related effect was controlled by recruiting subjects from two age groups: young (18 to 35 years, n = 40; Table 1, mean age: 23.90±0.43 years) and middle-aged adults (40 to 55 years, n = 40; Table 1, mean age: 47.15±0.72 years). Recruitment commenced in July 2014 and was completed in May 2015. We did not register this study as a clinical trial before the enrolment of participants started, because we could not foresee any potential safety issue. We registered this study retrospectively at ClinicalTrials.gov after we were reminded that our study design indeed met the WHO’s definition of a clinical trial. We confirm that all ongoing and related trials for this intervention are registered.

**Table 1:** Demographic information and baseline data (mean±SE) of young and middle-aged participants.

Parameters	Young adults (18-35y, n = 40)	Middle-aged adults (40-55y, n = 40)	p-value
Age (y)	23.9±0.43	47.15±0.72	<0.001
Refractive error			
Spherical power	−2.71±0.28	−2.44±0.34	0.55
Cylindrical power	0.63±0.10	0.55±0.08	0.53
Total error score	32.40±3.22	79.80±7.14	<0.001
log contrast sensitivity score			
without glare	1.78±0.01	1.73±0.02	0.03
with glare	1.66±0.03	1.50±0.03	0.001

All participants used the computer for over 2 hours every day. Exclusion criteria included: best corrected visual acuity worse than LogMAR 0 in either eye, history of ocular diseases and surgeries, and abnormal colour vision based on the Ishihara colour vision test. All experimental procedures were approved by the ethics committee of The Hong Kong Polytechnic University (reference number: HSEARS20140512001-03) and were conducted according to the principles expressed in the Declaration of Helsinki. Written informed consent was obtained from the participants, and all tests were conducted in the optometry clinic of The Hong Kong Polytechnic University.

#### General procedures

In the first clinic visit, subjective refraction was conducted with maximum-plus-maximum-acuity as the endpoint.[41] Using the same spectacle frame pre-adjusted for each individual, three pairs of single-vision spectacle lenses differing in tinted properties (i.e., two blue-light filtering lenses: BF and BT; one control lens: AR) were prescribed. For young adults, we prescribed the lenses based on the full distance prescription. For middle-aged presbyopic participants, an extra plus lens power (+0.50 to +2.00D) needed for comfortable near vision, as determined by the Percival’s mid-third criterion,[42] was added to the distance prescription.

In the next three visits (visits 2–4), one of three pairs of spectacle lenses was delivered with its identity hidden. The sequence of lens types was pseudo-randomized for each individual, i.e., participants were allocated in different sequences of lens wear by the date of admission. While wearing the new pair of lenses, the participants performed contrast sensitivity and colour discrimination tests using two common clinical tests: the Mars contrast sensitivity chart and Farnsworth Munsell 100 hue test. All participants were asked to wear the assigned spectacles for one month for a minimum of 2 hours per day. After each monthly wearing period (visits 3–5), the participants’ lens performance, night vision quality, and sleep quality were assessed subjectively using a questionnaire (S1 Table, scoring from 1 [very unsatisfactory] to 5 [very satisfactory]). At the end of the study, the participants were asked to choose their preferred lens type among the three pairs of lenses.

#### Contrast sensitivity with and without glare

It was originally proposed to use csv-1000 contrast sensitivity test (VectorVision, US). This test should be performed at 2.5m as recommended by the manufacturer; however, most of the middle-aged presbyopic participants required more than +0.50D additional power (i.e., far point < 2.0m) for clear computer vision. We therefore used the Mars contrast sensitivity letter chart to test contrast sensitivity (Mars Perceptrix, Chappaqua, NY).[43, 44] One of three charts differing in their letter combinations was selected randomly in order to avoid memorization of the charts. The chart was placed at 50 cm with each letter subtended 2° visual angle. Each chart consisted of 48 contrast levels, ranging from 91.2% to 1.2%. The contrast of each subsequent letter was decreased by a factor of 0.04 log unit. Followed the recording procedures as specified by the manufacturer, participants were instructed to read the letters from high to low contrast and the test ended when two consecutive errors were made. The contrast sensitivity was scored as the log contrast sensitivity of the last correct letter minus 0.04 log unit for every prior error. The test was administered under normal (room illumination, 400 lux) and glare conditions. A brightness acuity tester set at its medium light intensity level (100 foot lamberts) simulated the glaring condition. The clinical significance level was defined as 0.11 log unit based on the standard deviation obtained in normal subjects. [43]

#### Colour discrimination

The Farnsworth Munsell 100 hue test (X-Rite, USA) was used to evaluate colour vision. This test consists of four trays each containing 21 movable caps. Participants were asked to sort the randomly arranged caps following the hue order from the first to the last fixed caps. The total error score was calculated, as documented in the instruction manual, to quantify the accuracy of colour discrimination. [45] The clinical significance level was defined as 29 total error score, based on the standard deviation obtained in normal subjects. [45]

### Statistical analysis

A prior power analysis estimated a total sample size of 66 to produce 80% power (effect size = 0.20, α level = 0.05, power = 0.80, number of groups = 2, number of measurements = 3) to detect within-group difference for the primary outcome measures (i.e., log contrast sensitivity and total error scores for colour discrimination). Assuming a 45% of dropout rate, the original protocol aimed to recruit 120 participants. However, because of the difficulty in recruiting enough participants within a short recruitment period, only 82 participants were recruited. Of these participants, 80 completed the entire experiment, yielding 95% power.

#### Clinical visual performances

All statistical analyses on clinical visual performances were two-tailed and performed using SPSS 23 (IBM, USA), with a significance level set at α<0.05. An unpaired t-test was performed to determine any baseline differences between the two age groups. To analyze the contrast sensitivity (log contrast sensitivity score with and without glare) and colour discrimination (total error score) data, a 2 (age groups) X 3 (lens types) two-way mixed ANOVA was performed with age groups (young and middle-aged adults) being the between-participants factor, and lens types (BF, BT and AR lenses) being the within-participant factor. Mauchly test indicated that the assumption of sphericity was not violated (all *X*^*2*^*<2*.*50*, p>0.25). Although the data violated the assumption of normality (Kolmogorov-Smirnov tests, all p<0.05), the balanced samples with reasonable sample sizes (n = 40 per age group) should not significantly affect the false positive rate.[46] The analysis was then repeated using non-parametric tests which confirmed there was no significant effect of the lens on the two clinical visual performances (Friedman tests, all p>0.15).

#### Lens performance questionnaire

All statistical analyses on lens performance questionnaire were performed using Minitab 15 (Minitab Inc., USA), with the significance level set at α<0.05. First, a Friedman test compared the questionnaire scores of different lenses (items related to outdoor activities and night vision in middle-aged adults were excluded because their prescriptions were not prescribed for night-time outdoor use). Then, the questionnaire data from the two age groups was combined and the number of participants who reported “improvement” (score of BF or BT > score of AR), “decline” (score of BF or BT < score of AR), or “no change” (score of BF or BT = score of AR) in lens performance when wearing the blue-light filtering spectacle lenses was counted. One-sample Chi-square test was used to determine whether the proportion of these three responses (i.e., “improvement”, “decline”, and “no change”) was evenly distributed.

## Results

### Blue light transmission characteristics

**Fig 2:**
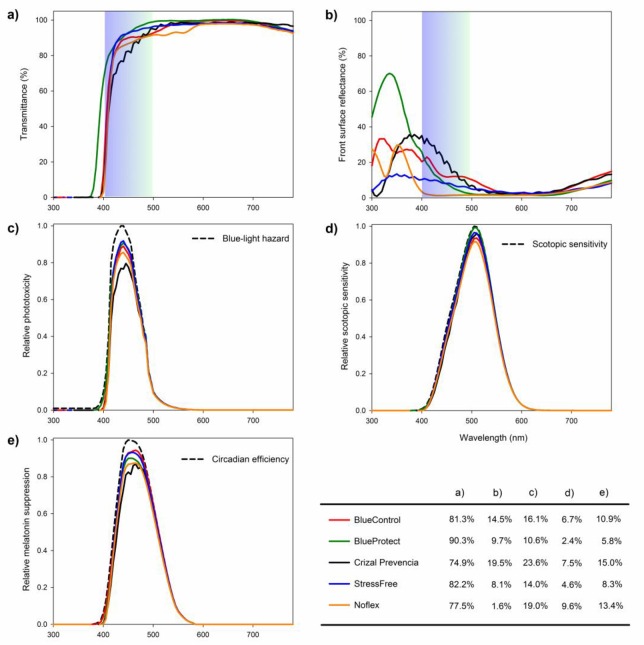
**The spectral transmittance (a), front surface reflectance (b), phototoxicity (c), scotopic sensitivity (d), and melatonin suppression (e) as a function of wavelength for the five blue-light filtering spectacle lenses.** The standard action spectrum curves for the blue-light hazard, scotopic luminous efficiency and circadian efficiency are displayed as dashed lines. The data are summarized in the table at the bottom right.

The spectral transmittance curves for each of the five lenses are shown in Fig 2A. All lenses almost completely blocked UVA, UVB, and near-UV (380–400 nm) rays (maximum transmittance: 0.12%). Blue light especially near the end of the visible light spectrum was filtered (blue-light transmittances: 74.9% to 90.3%), while a relatively high transmission for visible lights (400–780 nm; transmittance: 86.1% to 93.2%) was maintained. BlueControl, BlueProtect, Crizal Prevencia, and StressFree reduced blue-light transmission mainly by surface coatings (Fig 2B, blue-light reflectance: 8.1% to 19.8%). Noflex had a regular anti-reflection coating on the front surface (Fig 2B, blue-light reflectance: 1.6%) and reduced blue-light transmission mainly by absorption through brown-tint materials. In summary, blue-light filtering spectacle lenses theoretically reduced the calculated phototoxicity by 10.6% to 23.6% (Fig 2C). However, reduction in blue-light transmission sacrificed 2.4%-7.5% of light sensitivity under a dark environment (Fig 2D) and caused a small decrease in melatonin suppression efficiency of 5.8%-15% (Fig 2E).

### Clinical visual performances

Eighty participants completed the study. The baseline clinical data measured with their habitual spectacles are listed in Table 1. The two age groups did not differ significantly in refractive error (spherical and cylindrical power: unpaired t-test, *t≤0*.*60*, *df = 74*, *p≥0*.*33*). As expected, young adults had higher contrast sensitivity (with or without glare) and better colour discrimination ability than middle-aged adults (unpaired t-test, *t≤–2*.*26 or t = 6*.*05*, *df ≥ 54*, *p≤0*.*03*).

**Fig 3:**
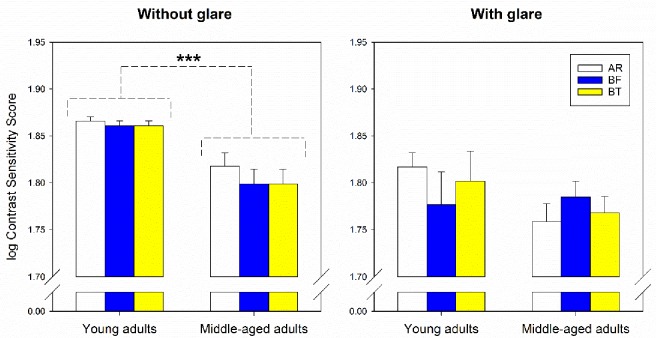
Log contrast sensitivity scores (mean ±SE) in normal (left) and glare conditions (right) when wearing different tinted lenses in young and middle-aged adults. AR, white bars. BF, blue bars. BT, yellow bars. There was a significant difference in contrast sensitivity under the no-glare condition between the two age groups: *** p<0.001.

**Fig 4:**
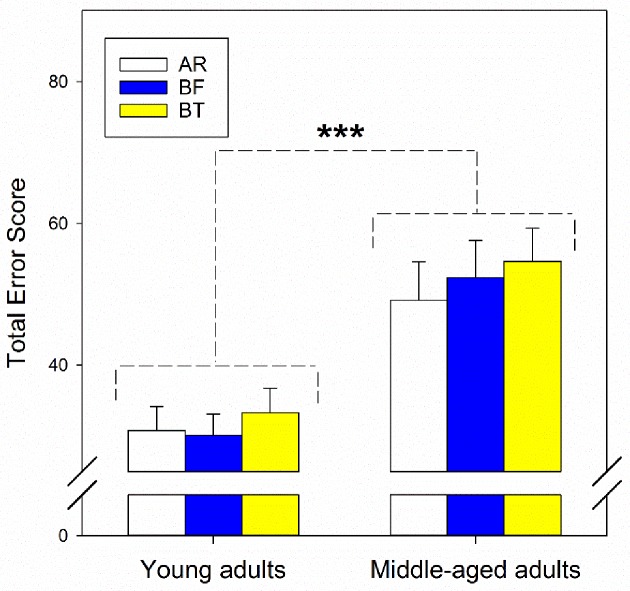
Total error scores (mean ±SE) when wearing different tinted lenses in young and middle-aged adults. AR, white bars. BF, blue bars. BT, yellow bars. There was a significant difference in total error scores between the two age groups: *** p<0.001.

Statistical analysis revealed that the two blue-light filtering lenses had no significant negative impact on contrast sensitivity, in both glare and without glare conditions, or colour vision. Although the middle-aged adults had significantly lower log contrast sensitivity scores in normal condition (Fig 3, without glare, *F*_*1*,*78*_ *= 18*.*79*, *p<0*.*001*, *η*^*2*^ *= 0*.*32*) and higher total error scores (Fig 4, *F*_*1*,*78*_*≤14*.*12*, *p<0*.*001*, *η*^*2*^ *= 0*.*44*) than young adults, there was no statistically significant interaction between age and lens types (*F*_*2*,*156*_*≤1*.*10*, *p≥0*.*34*, *η*^*2*^*<0*.*02*) or lens effect for both linear and quadratic trends (*F*_*2*,*156*_*≤3*.*17*, *p≥0*.*08*, *η*^*2*^*<0*.*02*) in log contrast sensitivity scores with (95% confidence intervals [CI]: AR–BT [–0.05, 0.05], AR–BF [–0.05, 0.06], BT–BF [–0.06, 0.06]) or without glare (95% CI: AR–BT [–0.01, 0.03], AR–BF [–0.01, 0.03], BT–BF [–0.02, 0.02], Fig 3) and total error scores (95% CI: AR–BT [–9.07, 1.02], AR–BF [–7.06, 4.46], BT–BF [–3.12, 8.57], Fig 4). A post-hoc power analysis was conducted using G*Power software.[47] With the current sample size (n = 80), the analysis revealed that the statistical power (≤0.42) for detecting a small effect (Cohen’s f = 0.10) in contrast sensitivity tests was weak. However, this study has sufficient power (>0.80 [48]) to detect medium (f = 0.25) to large effect size (f = 0.40) in contrast sensitivity tests and small to large effect size in colour discrimination test.

### Lens performance questionnaire

**Fig 5:**
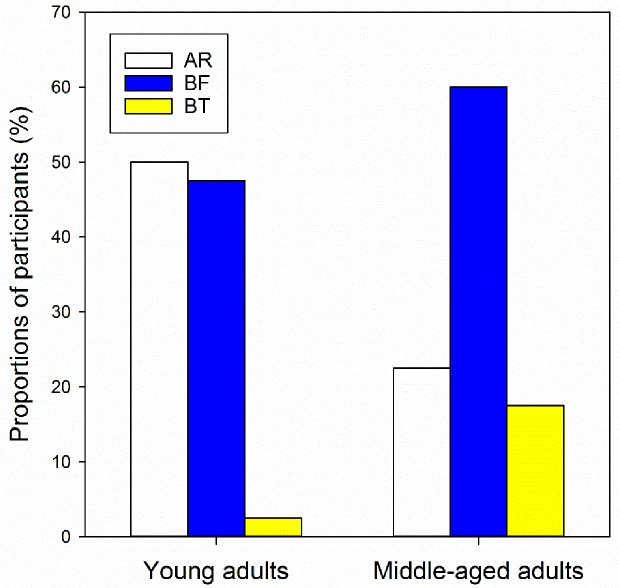
Participants’ preference of lens type in young and middle-aged adults. AR, white bars. BF, blue bars. BT, yellow bars.

A lens performance questionnaire was collected at the end of each wearing period. No significant difference was found in sleep quality and lens performance in middle-aged participants (Friedman test, all *p>0*.*05*). However, young adults were less satisfied with the BT lens due to the problems related to colour contrast, colour discrimination, and lens appearance when compared to AR and BF lenses (Friedman test, all *p<0*.*05*). Additionally, participants’ preferences of lens type were significantly different between the two age groups (Fig 5, Chi-square test, *DF = 2*, *X*^*2*^ *= 9*.*92*, *p = 0*.*007*). Young adults preferred the AR (n = 20, 50.0%) and BF (n = 19, 47.5%) lenses compared to the BT lens (n = 1, 2.5%). However, the middle-aged adults preferred the BF lens (n = 24, 60.0%) to the other two lens types (AR n = 9, 22.5%; BT n = 7, 17.5%).

**Fig 6:**
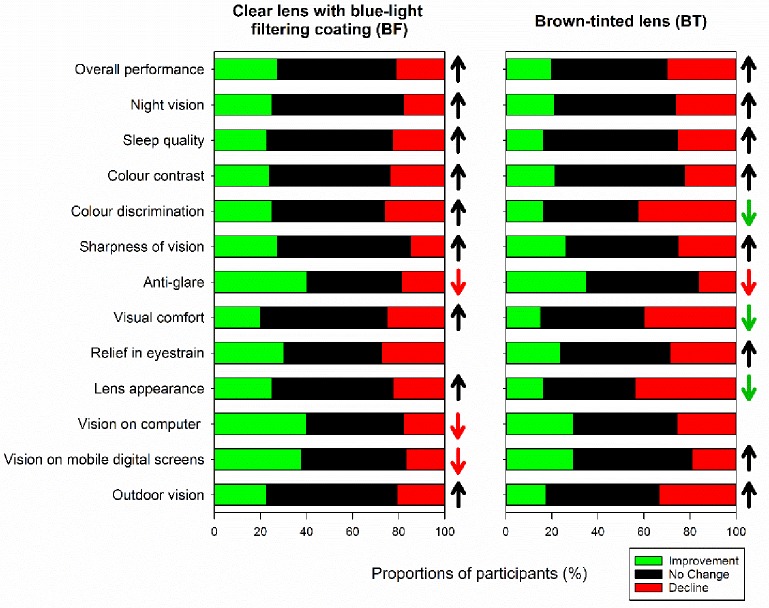
The proportions of participants showing “improvement”, “no change” and “decline” in lens performance when wearing BF (left) and BT (right) lenses. The arrows indicate questionnaire items showing significantly diverse responses for each item (One-sample Chi-square test, all *p<0*.*05*). The colours of the arrows denote the response that contributed most to the Chi-square test (i.e., highest *mini-X*^*2*^). For example, upward black arrows indicate that the diverse responses revealed in Chi-square tests were due to high proportions of “no change”; downward green and red arrows indicate that the diverse responses were due to low proportions of “improvement” and “decline”, respectively.

We further investigated whether our participants observed changes in lens performance when wearing blue-light filtering spectacle lenses. One-sample Chi-square tests showed significant differences between the proportion of “improvement”, “decline”, and “no change” responses in all (Fig 6, all *X2>7*.*50*, *p<0*.*03*) except two questionnaire items (*relief in eyestrain* for BF lens and *vision on computer* for BT lens, both *X2<5*.*00*, *p>0*.*05*). The response that had the highest *mini-X2*, i.e. the highest contribution to the chi-square test statistic was then identified. For most questionnaire items, the diverse responses were mainly due to the high proportions of participants who perceived “no change” in lens performance (>45%, Fig 6, black bars and arrows, all *mini-X2 > 3*.*70*). On the other hand, the diverse responses in *anti-glare* performance when wearing BF and BT lenses and *vision on computer* and *vision on mobile digital screens* when wearing BF lens were due to the low proportions of “decline” responses (<19%, Fig 6, red bars and arrows, all *mini-X2>5*.*00*). Conversely, the diverse responses in *colour discrimination*, *visual comfort*, and *lens appearance* when wearing BT lens mainly originated from the low proportions of “improvement” responses (<17%; Fig 6, green bars and arrows, all *mini-X2>7*.*00*).

## Discussion

While maintaining sufficient visible light for twilight and night driving (>80%),[37] all five blue-light filtering lenses chosen in this study reduced blue light transmission. This category of filtering lenses, including spectacle lenses and intraocular lenses, is proposed as a protective measure against blue-light hazard.[18, 23, 49, 50] Laboratory studies have confirmed that reducing blue-light (430 nm) transmission through a blue-light filter by 50% could reduce approximately 80% of photochemical damage to the retina.[22] However, AMD is a multi-factorial eye disease, which has risk factors including age, smoking, nutritional status, sunlight exposure and genetic background [10–12, 14, 51, 52]. The disease takes years to develop and progress. It is difficult to directly comprehend the protective efficacy of the blue-light filtering lenses in human eyes. In this study, the theoretical protective efficacy of these lenses was calculated based on established standards that took blue-hazard function and lens spectral transmittance data into account (see methods). The calculation indicates that blue-light filtering spectacle lenses theoretically reduced 10.6% to 23.6% of the potential phototoxicity by blocking the hazardous radiation between 400 to 500 nm. Because these spectacle lenses are non-invasive and easy to prescribe and handle, the availability of similar products in the market is expected to increase. Our data can serve as a useful reference for future lens design.

Although overexposure to blue light is phototoxic, blue light is essential for normal night vision and circadian rhythms. One concern of using blue-light filters as a protective measure against blue light hazard[31, 33, 53] is that the peak sensitivity for night vision and melatonin suppression is within the blue-green and blue spectrum, respectively. To understand how the blue-light filtering lenses affect visual and physiological functions, the relative change in scotopic sensitivity and melatonin suppression was first calculated based on their spectral transmittances. These lenses slightly attenuate scotopic sensitivity and melatonin suppression by 2.4%-7.5% and 5.8%-15.0%, respectively. Two of the five blue-light filtering spectacle lenses were then tested in a pseudo-randomized control clinical trial, in which the wearers were asked to judge whether their night vision and sleep quality were affected after a one-month wearing period. Most participants reported no change in lens performance compared to the control lens. It should be noted that the result for night vision might not be applicable to the middle-aged participants as their prescriptions were mainly for computer use, rather than night-time outdoor use. In agreement with our findings, other studies on intraocular lenses also concluded that blue-light filters did not have a remarkable impact on vision in dark environments[25, 54] or on sleep quality[29]. It is worth noting that such intraocular lenses block more blue light than any of the spectacle lenses tested in the present study and theoretically reduce scotopic sensitivity by as much as 20%.[53]

In addition, contrast sensitivity with and without glare, and colour vision of the lens wearers was evaluated and it was found that the two blue-light filtering spectacle lenses did not significantly affect these two visual functions. Similar results have been previously reported for blue-light filtering intraocular lenses.[24–28, 30, 54, 55] It has been reported that dense optical filters can enhance contrast sensitivity under glare conditions,[49] but degrade colour vision.[20] In general, our results suggest that blue-light filtering lenses with more than 70% of blue-light transmission do not markedly affect visual performance.

In this study, the crossover study design allowed a direct comparison among the three lenses for each individual participant. This avoided potential inter-subject variability that could arise if the lens effects are assessed by comparing different groups of subjects. We controlled subjective bias by allocating the lenses in a pseudo-random sequence with their identities masked, although the slight tinted appearance of the BT lens might have been easier to identify.

In brief, the blue-filtering lenses modestly filter high energy, short-wavelength light and do not markedly degrade visual performance. More than one-third of wearers found that a clear lens with blue-filtering coating (BT lens) provided better anti-glare performance and improved their vision for computer and mobile digital screens (Fig 6). This type of blue-light filter could serve as a supplementary option for protecting the eye from potentially harmful blue light. Our data could be used as a framework for future lens design.

## Supporting Information

S1 Checklist CONSORT checklist. This includes the CONSORT 2010 checklist.
(DOC)

S1 Data Raw data. This includes the raw data.
(XLS)

S1 Logfile SPSS log file. The log file lists the statistics used for visual performance analyses using SPSS.
(TXT)

S2 Logfile Minitab log file. The log file lists the statistics used for questionnaires analyses using Minitab.
(TXT)

S1 Protocol Original proposal. This is the original proposal for human ethics application.
(DOC)

S2 Protocol CONSORT flow diagram. The flow diagram describes the experimental progress.
(DOC)

S1 Table Questionnaire designed to evaluate the effects of blue-light filtering lenses on visual performance and circadian rhythm. The original version is written in Chinese.
(DOCX)
