# Supplementary material for: Blue-Light Filtering Spectacle Lenses: Optical and Clinical Performances
Source: PLoS One. 2017 Jan 3;12(1):e0169114. doi: 10.1371/journal.pone.0169114 (PMC5207664; doi:10.1371/journal.pone.0169114)
Supplement: S1 Protocol — This is the original proposal for human ethics application. (DOC) [file pone.0169114.s005.doc]

**Effects of blue-light blocking lens on visual functions**

**Abstract**

**Working Hypothesis:** The blue-light blocking lens has no effect on the contrast sensitivity, accommodative response, color vision, and subjective grading of the quality of life and vision.

**Purpose:** To determine and compare the visual performances after wearing the blue-light blocking lenses in participants with and without presbyopia.

**Methods:** One hundred and sixty computer users (computer usage >2 hours/day) with (n=120, aged >40years) and without presbyopia (n=40, aged 18-35 years) will be recruited. Three pairs of single-vision ophthalmic lenses will be prescribed for the participants, in which one pair of them will be the blue-light blocking lenses (StressFree, Swisscoat, HK). The lenses will be used for intermediate vision (i.e., computer usage) in presbyopic group, and for distant vision in non-presbyopic group. The adaptation period for each pair of lenses will be 1 month. The pre- and post-treatment contrast sensitivity, accommodative response and color vision will be measured. The participants will also be asked to complete questionnaires about their quality of life and vision, and the performances of these ophthalmic lenses.

**Significance:** The blue-light blocking ophthalmic lens reflects the short-wavelength lights and protects the retina from “blue-light hazards”; however, blue lights are essential for various visual functions and circadian rhythms. This study will evaluate the impact of the blue-light blocking lenses on visual function and quality of life.

This proposal is the version that was submitted to and approved by the ethics committee before the trial began.

**Background**

Within the visible spectrum (380 to 780nm), the blue lights comprise the shortest wavelength radiations (380 to 500nm) and carry the highest amount of energy. Long exposure to these high energetic radiations – the blue light hazards – may damage the retinal structures and increase the risk of age-related macular degeneration.^1–3^ In our daily life, the blue lights appear in everywhere, e.g., from the sun, light bulbs and the electronic devices, but the conventional ophthalmic lenses (even with regular coatings) is unable to filter out these potentially harmful radiations. On the other hand, the recently launched blue-light blocking lenses reflect the short-wavelength radiations and provide an extra protection from the “blue-light hazards”.

Despite of the potential damages to the retina, the blue lights are important for normal visual functions, such as color vision^4^ and accommodation^5,6^. Moreover, blue lights may regulate the circadian rhythms by controlling the melatonin secretion.^7,8^ Thus, it is necessary to investigate the impacts of the blue-light blocking lenses on various visual function and quality of life. The aim of this study is to determine and compare the contrast sensitivity, accommodative response, color vision, and subjective grading of the quality of vision and life after wearing the blue-light blocking lenses in participants with and without presbyopia.

**Methods**

**Study design:** Randomized-control repeated measures

**Participants:**

One hundred and sixty computer users (computer usage >2 hours/day) with (n=120, aged >40years) and without presbyopia (n=40, aged 18-35 years) will be recruited. Participants with visual acuity worst than 0 logMAR, abnormal binocular and color vision, and previous history of ocular surgeries and pathologies will be excluded. The dilated fundus examination, fundus photography, slit-lamp examination, corneal topography, and macular pigment density measurement will be carried out to identify any ocular anomalies. Three pairs of single-vision ophthalmic lenses will be prescribed: in the presbyopic group, the ophthalmic lenses will be prescribed for computer use (intermediate working distance) and the eligible participants will be advised not to use the new lenses for outdoor activities; in the non-presbyopic group, the ophthalmic lenses will be prescribed for distant vision and the eligible participants could wear the lenses all day.

In the presbyopic group, the tentative additional power for intermediate working distance, which depends on the participant’s own working habit, will be measured using the binocular crossed-cylinder technique, followed by the adjustment within the Percival’s Mid-third criterion. Only those who require at least +0.50D of additional power for computer usage will be invited to participate.

Each participant will receive three pairs of ophthalmic lenses with different coatings and color – Presbyopic group: 1) clear anti-reflective (AR) regular coating, 2) regular AR coating with yellow tint, and 3) blue-light blocking coating with yellow tint (StressFree 32, 38, or 38B coating); Non-presbyopic group: 1) clear AR regular coating, 2) regular AR coating with yellow tint, and 3) clear blue-light blocking coating (StreeFree coating). Two clear lenses are chosen for the non-presbyopic group, because they are more cosmetically suitable for outdoor activities. Tinted lenses are suggested, although still under debates, to improve contrast^9^ and reading performances^10,11^; they are included to comparing the visual performances with the clear lenses and blue-light blocking lenses. All participants will be advised to wear the new ophthalmic lenses at least 2 hours per day. In the presbyopic group, the three blue-light blocking coatings will be randomly assigned. A double-blinded procedure will be applied, at least for the two yellow-tint lenses in the presbyopic group and the two clear lenses in the non-presbyopic group.

**Lab measurements:**

The csv-1000 contrast sensitivity test (VectorVision, US) will be used to measure the contrast sensitivity function, at spatial frequency of 3, 6, 12 and 18cpd, with and without glare. The color vision will be assessed by Farnsworth Munsell 100 hue test and the error-scores will be used for data analysis. The Royal Air Force rule will be used to measure the amplitude of accommodation.

**Procedures:**

The whole experiment will be divided into 5 visits (Figure 1). In Visit 1, we will measure the participants' visual acuity, refractive error, contrast sensitivity, accommodative response, color vision and ocular health. All participants will be asked to complete a questionnaire about their quality of life and vision. The eye examination will take about 1.5 to 2 hours in the Optometry clinic (AG048) of the Hong Kong Polytechnic University. They will be asked to prepare their own spectacle frame and three pairs of ophthalmic lenses will be prescribed. A deposit of $500 will be charged for the lenses, which will be refunded if they complete all 5 visits.

In Visits 2, 3 and 4, one of the three prescribed ophthalmic lenses will be randomly delivered. In each visit, the participants’ contrast sensitivity, accommodative response and color vision will be measured. The measurements will take about 45 minutes. In Visit 3, 4 and 5, the participants will be asked to complete a questionnaire about the performance of the ophthalmic lenses after 1-month adaptation. The deposit will be fully refunded in Visit 5.

Estimated duration: 9 months

Analyses:

1. Comparisons of spectral transmittance and reflectance across the four lens coatings
2. Comparisons of the effects of blue-light blocking antireflection coating between groups
3. Comparisons of the effects of clear and yellow-tinted lenses between groups
4. Comparisons of the effects of blue-light blocking antireflection coating versus yellow tint in each group


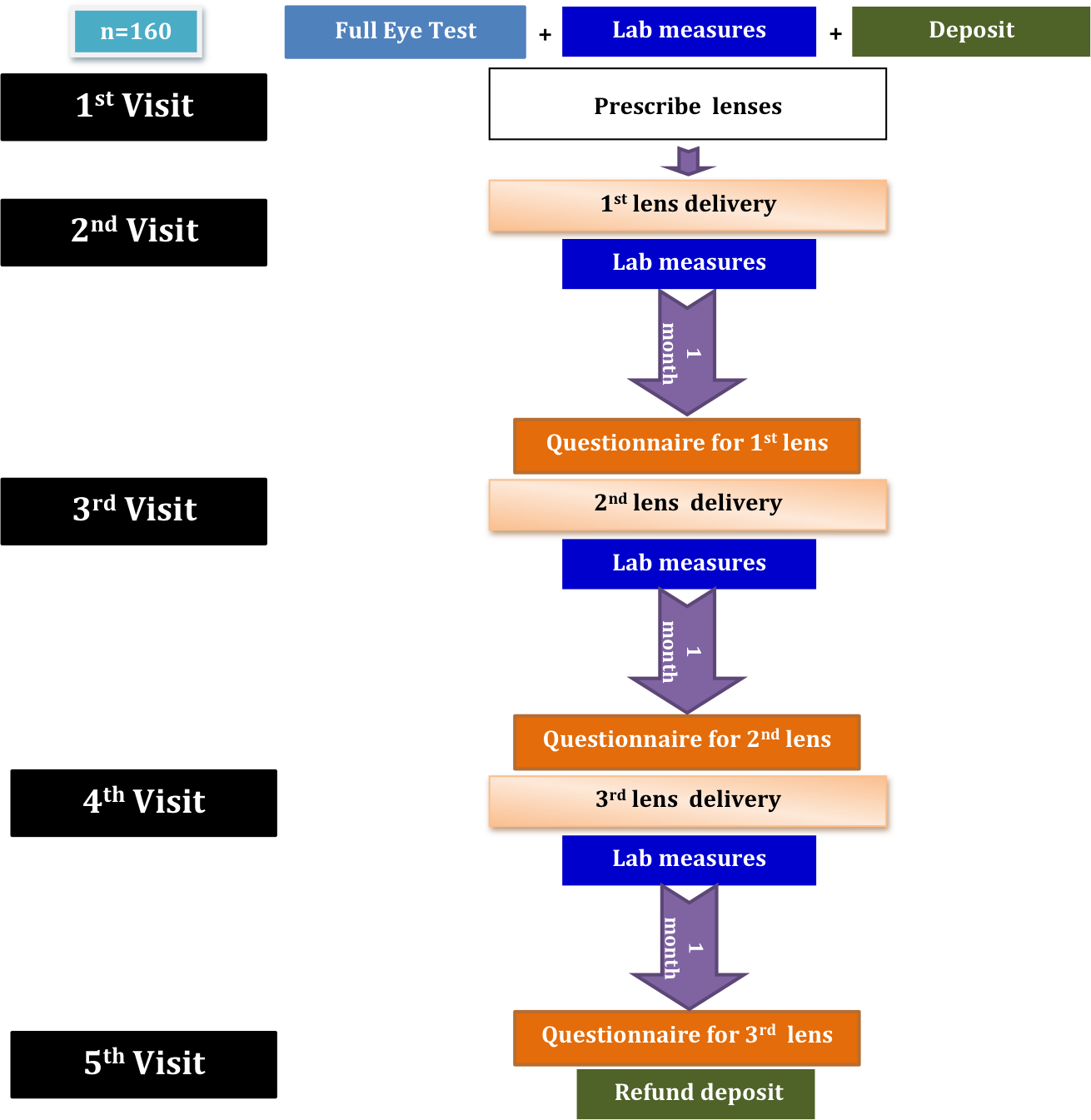


Figure 1. Flow chart for project’s milestones.

**References**

1. Wu J, Seregard S, Algvere PV. Photochemical damage of the retina. *Surv Ophthalmol*. 2006;51(5):461–481.

2. Taylor HR, Munoz B, West S, Bressler NM, Bressler SB, Rosenthal FS. Visible light and risk of age-related macular degeneration. *Trans Am Ophthalmol Soc*. 1990;88:163.

3. Ham WT, Mueller HA, SLINEY DH. Retinal sensitivity to damage from short wavelength light. 1976. Available at: http://www.nature.com/nature/journal/v260/n5547/abs/260153a0.html. Accessed May 12, 2014.

4. Wald G. The Receptors of Human Color Vision Action spectra of three visual pigments in human cones account for normal color vision and color-blindness. *Science*. 1964;145(3636):1007–1016.

5. Aggarwala KR, Nowbotsing S, Kruger PB. Accommodation to monochromatic and white-light targets. *Invest Ophthalmol Vis Sci*. 1995;36(13):2695–2705.

6. Kruger PB, Mathews S, Aggarwala KR, Yager D, Kruger ES. Accommodation responds to changing contrast of long, middle and short spectral-waveband components of the retinal image. *Vision Res*. 1995;35(17):2415–2429.

7. Dijk D-J, Archer SN. Light, sleep, and circadian rhythms: together again. *PLoS Biol*. 2009;7(6):e1000145.

8. Lockley SW, Brainard GC, Czeisler CA. High sensitivity of the human circadian melatonin rhythm to resetting by short wavelength light. *J Clin Endocrinol Metab*. 2003;88(9):4502–4505.

9. Yap M. The effect of a yellow filter on contrast sensitivity. *Ophthalmic Physiol Opt*. 1984;4(3):227–232.

10. Evans BJ, Drasdo N. Tinted lenses and related therapies for learning disabilities–a review. *Ophthalmic Physiol Opt*. 1991;11(3):206–217.

11. Lightstone A, Lightstone T, Wilkins A. Both coloured overlays and coloured lenses can improve reading fluency, but their optimal chromaticities differ. *Ophthalmic Physiol Opt*. 1999;19(4):279–285.
