# Supplementary figures and images for: Blue-Light Filtering Spectacle Lenses: Optical and Clinical Performances
Source: PLoS One. 2017 Jan 3;12(1):e0169114. doi: 10.1371/journal.pone.0169114 (PMC5207664; doi:10.1371/journal.pone.0169114)

**
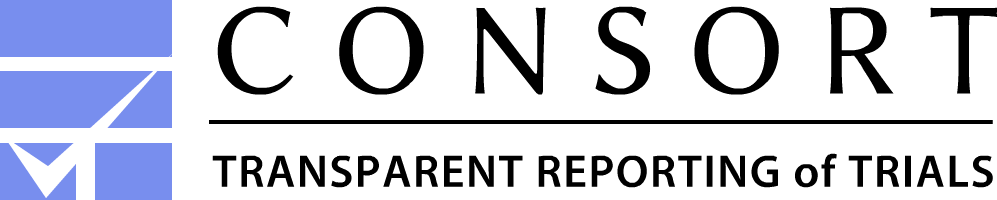
**

**CONSORT 2010 Flow Diagram**

**
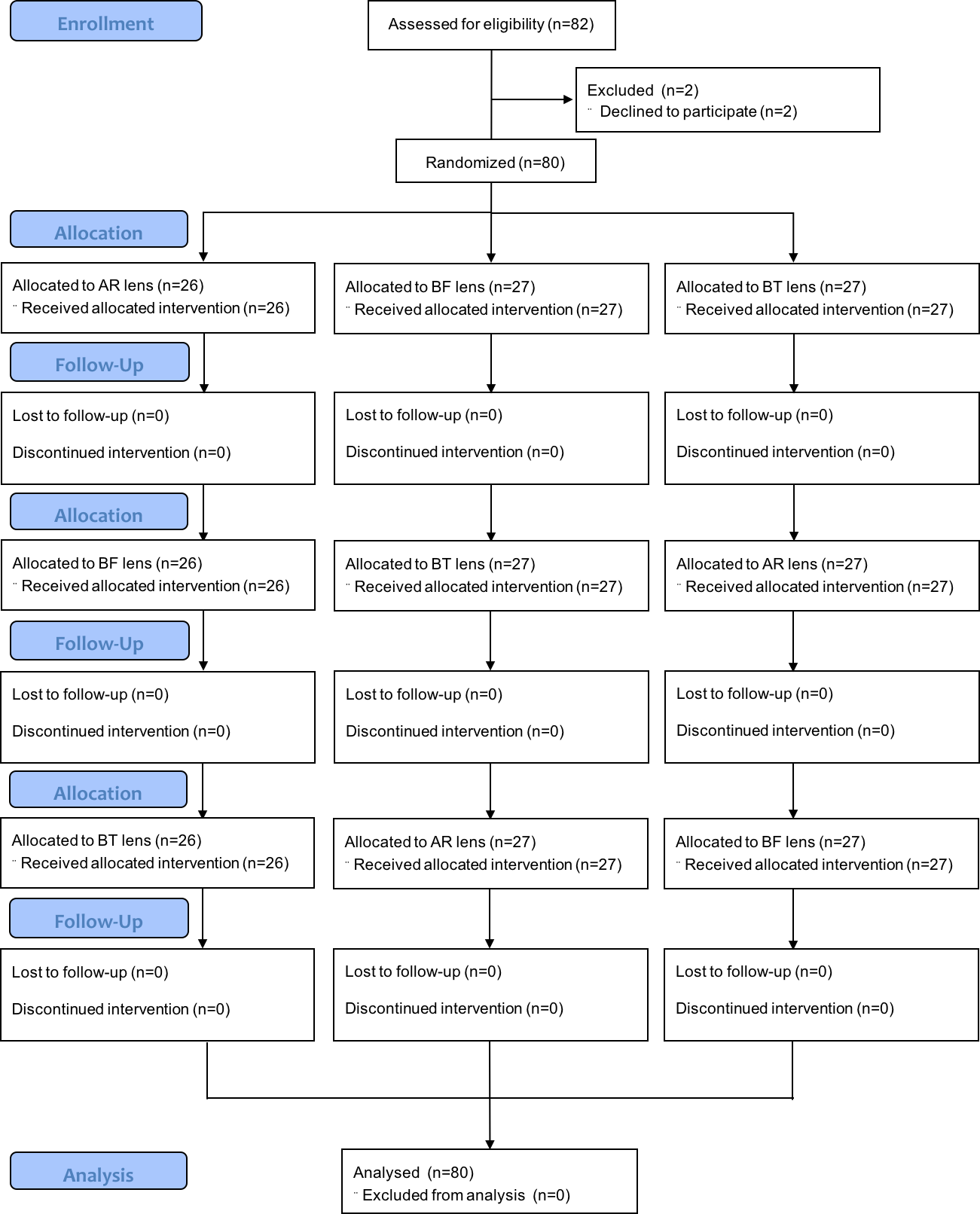
**

Supplement: S2 Protocol — The flow diagram describes the experimental progress. (DOC) [file pone.0169114.s006.doc]
