# Supplementary material for: Blue-Light Filtering Spectacle Lenses: Optical and Clinical Performances
Source: PLoS One. 2017 Jan 3;12(1):e0169114. doi: 10.1371/journal.pone.0169114 (PMC5207664; doi:10.1371/journal.pone.0169114)
Supplement: S1 Table — The original version is written in Chinese. (DOCX) [file pone.0169114.s007.docx]

**S1 Table**

Questionnaire designed to evaluate the effects of blue-light filtering lenses on visual performance and circadian rhythm. The original version is written in Chinese.

Please rate the performance of your new ophthalmic lenses:

|  | Very |  |  |  | Very |
| --- | --- | --- | --- | --- | --- |
|  | Unsatisfactory |  |  |  | Satisfactory |
| 1. Sharpness of vision | 1 | 2 | 3 | 4 | 5 |
| 2. Anti-glare | 1 | 2 | 3 | 4 | 5 |
| 3. Visual comfort | 1 | 2 | 3 | 4 | 5 |
| 4. Relief in eyestrain | 1 | 2 | 3 | 4 | 5 |
| 5. Colour contrast | 1 | 2 | 3 | 4 | 5 |
| 6. Colour discrimination | 1 | 2 | 3 | 4 | 5 |
| 7. Vision on computer | 1 | 2 | 3 | 4 | 5 |
| 8. Vision on mobile digital screens | 1 | 2 | 3 | 4 | 5 |
| 9. Lens appearance | 1 | 2 | 3 | 4 | 5 |
| 10. Sleep quality | 1 | 2 | 3 | 4 | 5 |
| 11. Outdoor vision* | 1 | 2 | 3 | 4 | 5 |
| 12. Night vision* | 1 | 2 | 3 | 4 | 5 |
| 13. Overall performance | 1 | 2 | 3 | 4 | 5 |

* If applicable
